# The Challenges of Vascular Implants: Regulatory Strategies and Biological Responses

**DOI:** 10.1002/smsc.202500379

**Published:** 2025-11-03

**Authors:** Serena Babboni, Rosa Sicari, Lara Russo, Virgilio Mattoli, Giuseppina Basta, Serena Del Turco

**Affiliations:** ^1^ Institute of Clinical Physiology CNR Via Moruzzi, 1 56124 Pisa Italy; ^2^ Center for Materials Interfaces Istituto Italiano di Tecnologia Via R. Piaggio 34 56025 Pontedera Italy

**Keywords:** biomaterials, heart failure, implantable vascular devices, inflammation, regulatory frameworks, thrombosis

## Abstract

Implantable vascular devices are becoming increasingly essential in clinical practice, particularly in the management of chronic cardiovascular diseases (CVDs), such as heart failure. These devices enable continuous hemodynamic monitoring, support early interventions, and promote personalized, cost‐effective care by providing real‐time data that enhance patient outcomes. However, their development and clinical application face significant regulatory and biological challenges. Regulatory frameworks, such as the European Union's Medical Device Regulation, ensure safety, efficacy, and high‐quality standards throughout a device's lifecycle. Despite these regulations, intravascular devices interact with vascular tissues and blood, triggering biological responses, such as inflammation and thrombosis, which may impair device functionality, reduce long‐term durability, and cause severe adverse events. The bioactive surface of implanted devices initiates inflammatory responses and coagulation, leading to complications like fibrotic encapsulation and vascular injury. After device implantation, endothelial injury promotes platelet activation, thrombus formation, and leukocyte infiltration, compromising both device integration and vascular function. Therefore, the material and structural design of these devices play a crucial role in mitigating thrombotic and inflammatory reactions. This review explores the potential benefits and challenges of vascular implantable devices in the management of chronic CVDs, highlighting regulatory aspects, biological responses, and future clinical perspectives.

## Graphical abstract

Implantable vascular devices are revolutionizing the management of cardiovascular diseases, such as heart failure, by enabling real‐time monitoring and individualized therapy. However, their potential is challenged by regulatory constraints and biological barriers, as device implantation into a vessel can trigger inflammation, thrombosis, and long‐term failure. This review outlines clinical potential, regulatory frameworks, and future challenges in this field.

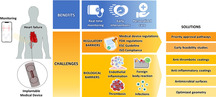

## 1 Introduction

Implantable vascular devices are increasingly used in clinical practice, playing a critical role in the management of chronic cardiovascular diseases (CVDs), such as heart failure (HF).^[^ ^1^, ^2^ ^]^ These devices enable continuous hemodynamic monitoring, support early intervention, and facilitate personalized, cost‐effective care.^[^ ^2^ ^]^ By providing reliable real‐time data, they not only complement standard therapeutic approaches but also significantly improve patient outcomes, as their performance impacts both overall health and quality of life (QoL).

However, the development and use of such devices encounter regulatory and biological challenges that require stringent regulatory assessments and preclinical understanding of the host's biological responses. A critical aspect is the regulatory strategy adopted to ensure the safety and efficacy of such devices. These devices must overcome stringent regulatory barriers before entering the clinical market. The medical device regulation (MDR) is the regulatory framework in the European Union (EU) designed to improve patient safety and ensure that medical devices meet high standards of quality, safety, and performance throughout every stage of the lifecycle.^[^ ^3^ ^]^

Implanted intravascular devices interact with vascular tissues and blood, triggering a cascade of biological responses such as inflammation, thrombosis, or infections that can impair functionality, reduce long‐term durability, and lead to severe adverse events.^[^ ^4^, ^5^ ^]^ Once implanted, the device presents a bioactive surface to circulating blood components, facilitating inflammatory cell adhesion, activation, and initiating coagulation, contributing to vascular injury.^[^ ^5^ ^]^ The immune system may recognize the device as a foreign body, initiating acute or chronic inflammatory responses that can lead to fibrotic encapsulation and adverse tissue remodeling. The endothelium plays a key role in maintaining vascular homeostasis, preventing thrombosis, modulating inflammation, and regulating vascular tone.^[^ ^6^ ^]^

**Figure 1:**
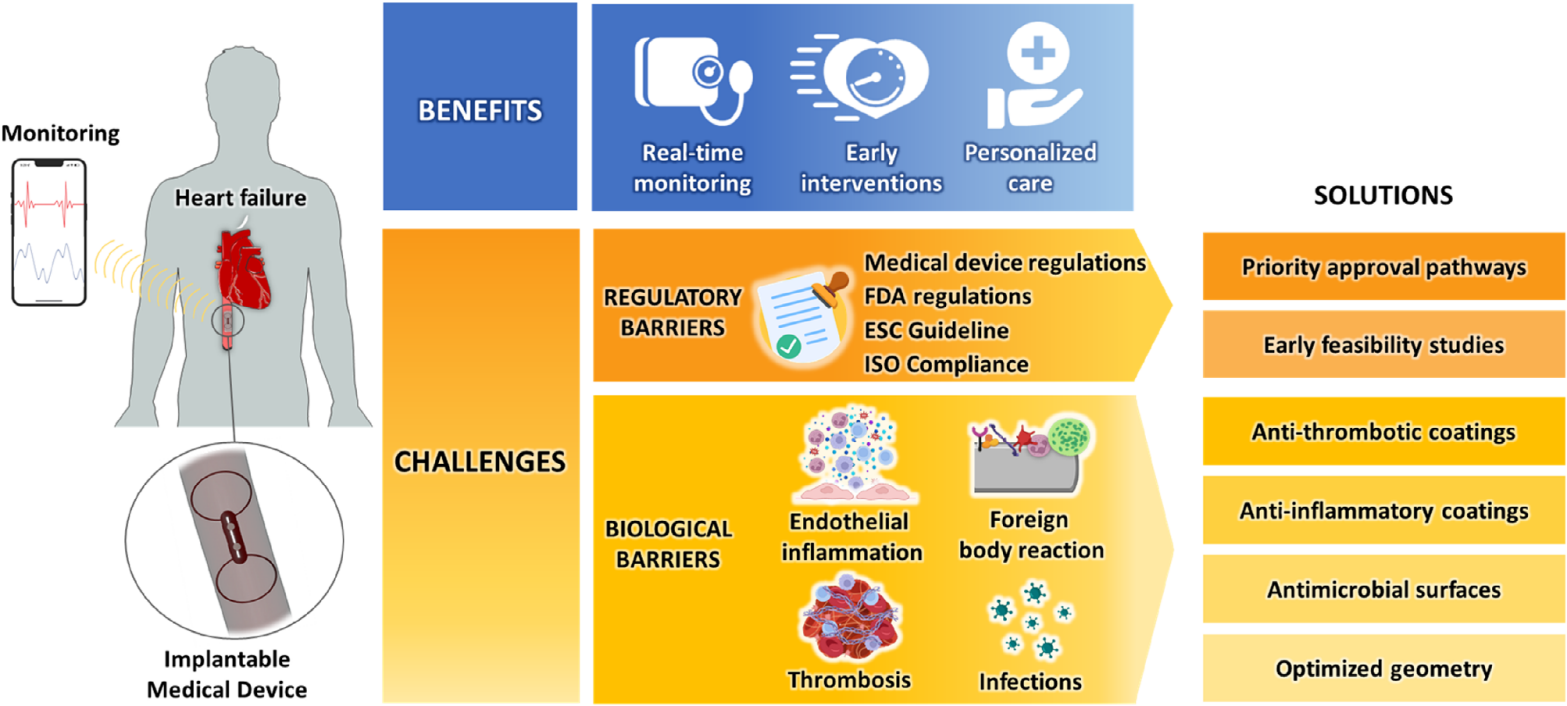
Clinical impact and translational challenges of implantable vascular devices for HF. FDA; ESC; ISO.

The implantation or exposure to altered hemodynamic flow patterns induced by the device induces injury to the vascular wall at the site of implantation.^[^ ^7^ ^]^ This damage promotes platelet activation, thrombus formation, expression of adhesion molecules, and leukocyte infiltration, which impair vascular function and device integration.^[^ ^5^ ^]^ Consequently, the biological assessment of the material and structural design of a device is a critical factor in medical device development to reduce adverse thrombotic and inflammatory reactions, infections, and ensure clinical efficiency. Together, these biological and regulatory challenges underscore the complexity of developing intravascular devices and highlight the need for interdisciplinary strategies that integrate material science, vascular biology, clinical expertise, and regulatory knowledge (**Figure** **1**).

This review highlights the potential benefits and challenges of vascular implantable devices in the management of chronic CVD, such as HF, offering an overview of the regulatory frameworks and biological responses, as inflammation and thrombosis, underlying the vascular‐device interaction.

## 2 Implantable Cardiovascular Devices: From Remote Monitoring to Personalized and Sustainable Care

Medical devices play a critical role in the management of CVDs, both in acute interventions and chronic care. Several commercially available medical devices are implanted in the heart, arteries (e.g., left ventricular assist device (LVAD), intra‐aortic balloon pump, stents, grafts), and veins (e.g., inferior vena cava filter, vein patch, or graft) to restore and maintain heart and vascular health.^[^ ^8^ ^]^

The progressive ageing of the global population is accompanied by a dramatic rise in the incidence of chronic diseases, which require long‐term monitoring of cardiovascular function to support early diagnosis, personalized therapy adjustments, and improved clinical outcomes. Implantable monitoring devices are dynamic systems designed to continuously measure and report key physiological parameters, such as pressure and hemodynamic data, in real time.

Recently, microelectromechanical system (MEMS)‐based sensor devices are increasingly used in implantable devices to monitor vital physiological parameters critical for the management and treatment of CVDs.^[^ ^9^, ^10^ ^]^ MEMS are miniaturized mechanical components (i.e., sensors and/or actuators) integrated with microelectronics. The ability to measure with high sensitivity a broad range of physical phenomena, such as temperature, pressure, inertial forces, chemical species, and radiation, makes MEMS a transformative technology with broad applications in healthcare.^[^ ^11^ ^]^ These devices collect data continuously, reducing the need for repeated invasive interventions and improving patient care. A noteworthy example is a MEMS‐based pressure sensor used in implantable devices such as cardiac pacemakers or HF management.^[^ ^12^ ^]^

### 2.1 Remote Monitoring of Patients with HF

The ability to detect heart issues early and respond with personalized interventions could significantly improve patient outcomes, especially for chronic conditions like HF. HF is a complex clinical syndrome due to reduced left ventricular ejection fraction (HFrEF) and is treated using a tiered medical therapy approach with good evidence of improved symptoms and life expectancy.^[^ ^13^ ^]^ Despite this, many patients remain symptomatic.^[^ ^14^ ^]^ Advanced HF is characterized by significantly impaired cardiac function and symptomatic limitation and traditional approaches to disease monitoring, such as periodic clinical visits and symptom‐based assessments, are insufficient to capture early signs of decompensation.^[^ ^14^ ^]^ For this, remote and continuous monitoring of HF patients has been a major task of device development over the last few years. Although no strategy has been proven to influence outcome, QoL and patients’ comfort have been improved. The possibility of observing changes in pressure, fluid volume overload, and heart rate before symptoms ensue is the mainstay of remote monitoring and its potential use in clinical practice. Up to 20% of ambulatory HFrEF patients still experience a HF hospitalization or die within 2 years and 50% of hospitalized HFrEF patients have a readmission within 6 months despite optimal medical therapy.^[^ ^13^, ^15^ ^]^ As such, there is an unmet need for better strategies in following up these patients to improve QoL and outcome.

The last decades have seen leaps in the use of devices in HF, either to provide monitoring with the aim of improved tailoring of care or directly providing therapy synergistic with medical therapies. Of all the potential clinical strategies, such as regular visits, phone calls, and wearable devices, none has been proven to meet patients’ needs and expectations. A document of the European Society of Cardiology (ESC) on future developments in the management of patients with stable HF recommends: “in order to reduce hospitalizations and mortality the use of multidisciplinary HF management programmes (HF‐MPs), which enable patients to have the correct investigations, an accurate diagnosis, appropriate evidence‐based therapy, education and suitable follow‐up. The optimal implementation of a HF‐MP requires a multidisciplinary team that is active along the whole HF trajectory—from onset, through critical events, periods of apparent stability, and its terminal stages”.^[^ ^16^ ^]^ In the gaps of evidence, the document lists the lack of studies on optimal models for the follow‐up of stable HF patients. The clinical needs open routes of technological developments to fill the gap in the management and treatment of HF patients.

The need for implantable monitoring devices in HF management is based on the chronic nature of the disease and the need for patient compliance with treatment to achieve long‐term objectives. A systematic *meta*‐analysis compared device‐based remote monitoring of congestion to standard therapy, demonstrating that a hemodynamic‐guided strategy with invasive devices is associated with a significant reduction in the composite endpoint of all‐cause death or HF compared to standard therapy.^[^ ^17^ ^]^

One of the most studied implantable devices in HF to monitor artery pressure is CardioMEMS, a MEMS‐based device that offers a cutting‐edge solution to HF management by enabling continuous, real‐time monitoring of key indicators like pulmonary artery pressure.^[^ ^18^ ^]^ The device has undergone rigorous evaluation in clinical trials and postmarketing surveillance to assess its safety profile.^[^ ^1^ ^]^ Increased pulmonary artery pressure values days and weeks before HF decompensation provide a window of opportunity for medical intervention to reduce pulmonary artery pressure, thus improving symptoms and limiting rehospitalization.^[^ ^1^ ^]^ The CHAMPION HF trial (CardioMEMS Heart Sensor Allows Monitoring of Pressure to Improve Outcomes in NYHA Class III HF Patients),^[^ ^1^ ^]^ the pivotal American multicentric study, demonstrated that its use was associated with reduced pulmonary artery pressures (PAPs) and fewer hospitalizations for HF. Furthermore, findings from the MONITOR HF Study,^[^ ^2^ ^]^ a European randomized multicentric trial involving CardioMEMS implantations, highlighted a high degree of sensor reliability, showing a benefit of hemodynamic monitoring in addition to standard care for patients with HF by substantially improving QoL and reducing HF hospitalizations.

Although adverse events are relatively rare, continued vigilance, and effective management strategies are essential to ensure the efficiency and safety of implantable devices associated with optimal patient outcomes.

### 2.2 Economic Impact and Personalized Care in Advancing Implantable Device Management

Implantable devices have transformed the treatment of both chronic and acute conditions, resulting in improved survival rates and a better QoL for patients.

Most studies on implantable vascular devices and HF have traditionally focused on clinical endpoints, such as mortality, hospitalization rates, procedural success, or device‐related complications. Conversely, few studies have systematically assessed QoL and other patient‐centered outcomes, despite their critical importance in evaluating the real‐world impact of these interventions. In fact, improvements in QoL and other patient‐reported outcomes represent a primary goal of HF management and are essential for guiding personalized care, optimizing treatment strategies, and ultimately enhancing the overall value of implantable therapies.^[^ ^13^ ^]^ HF management with CardioMEMS has been shown to reduce PAPs, improve functional status, QoL, and decrease HF hospitalizations at 1 year in real‐world, nonrandomized clinical trial populations.^[^ ^19^ ^]^ HF patients monitored with CardioMEMS over 24 months demonstrated sustained improvements in QoL across global and domain‐specific domains. Specifically, patients reported enhanced cognitive and emotional perceptions of their illness, high satisfaction with the monitoring device, and a strong adherence to remote hemodynamic data transmission.^[^ ^20^ ^]^ These benefits likely reflect the optimization of HF management through hemodynamic monitoring, which can prevent symptom progression and support patients in their daily activities.

The integration of implantable devices in clinical practice raises key questions about healthcare sustainability, highlighting the need to assess both economic feasibility and the personalized care needs. Cost‐effectiveness analyses are increasingly used to assess the balance between the initial costs of device implantation and the long‐term benefits, such as reduced hospital stays, fewer complications, and an improved QoL. Advances in biomaterials, miniaturization, and device lifetime have made these treatments more affordable by lowering maintenance costs and extending their functional lifespan. Importantly, cost‐effectiveness goes beyond financial considerations to include equitable access, patient safety, and personalized treatment options. The cost‐effectiveness varies considerably depending on the type of device, the selected patient group in which they are implanted, and the healthcare system or country context. About implantable devices to monitor HF patients, several studies have demonstrated that, despite its high upfront costs, the CardioMEMS offers long‐term savings by reducing hospitalizations and improving patient outcomes. Cost‐effectiveness analysis suggests that it is cost‐effective compared with usual care for patients with persistent New York Heart Association class III symptoms and at least one HF hospitalization within 12 months.^[^ ^21^, ^22^, ^23^ ^]^ When cost‐effectiveness is aligned with personalization, the benefits are synergistic: appropriate patient selection reduces the likelihood of device failure, unnecessary procedures, or overtreatment, while targeted follow‐up strategies optimize device performance and minimize downstream costs.

Personalized care represents the most impactful dimension in maximizing the clinical and economic value of implantable devices. Patient‐specific factors, such as age, comorbidities, genetic background, lifestyle, and risk of device‐related complications, must guide device choice, implantation approach, and follow‐up plans. While implantable devices are typically tested in carefully selected populations during clinical trials, patients in routine clinical settings often have multiple comorbidities that were not represented in these studies. Characteristics unique to each patient, including genetic background, diabetes mellitus (DM), obesity, renal problems, or infections, as well as individual vascular biomechanics, can influence the biological responses associated with the device. Individual genetic information could be useful in the management of patients with HF to tailor device choices and to reduce the development of complications.^[^ ^24^ ^]^ LVAD placement in HF patients is followed by thrombosis and bleeding complications, which are caused by high nonphysiologic shear stress and antithrombotic/anticoagulant therapy. A high risk of complications occurs in the presence of the genotype polymorphisms, which are involved in the coagulation system, hemostasis function, and metabolism of the therapy. Incorporating such genomic information into clinical practice may improve clinical outcomes with reduced LVAD complications in HF patients, fewer rehospitalization cases, which will be economically profitable for the healthcare system.^[^ ^25^ ^]^ DM is associated with impaired wound healing, accelerated atherosclerosis, and a proinflammatory status that enhances the vasculo‐proliferative response. Both elevated glucose levels and abnormal glycemic control are risk factors for surgical complications, making careful assessment crucial before medical device implantation, such as LVAD placement.^[^ ^26^ ^]^ However, there still is no literature consensus regarding the impact of DM on major adverse outcomes (pump thrombosis, stroke, infection, etc.) after LVAD implantation.^[^ ^27^ ^]^ In a comprehensive review, Kharbikar et al. discuss how implantable devices for treating diabetes face long‐term limitations due to the foreign body response (FBR), an immunological process which causes fibrosis and impairs device functionality. They emphasize that early modulation of the immune response, combined with tailored material strategies—such as surface modifications or anti‐inflammatory coatings—is critical to prevent fibrotic encapsulation. These insights provide a framework for designing personalized implants that consider patient‐specific inflammatory profiles and comorbidities, enhancing long‐term efficacy.^[^ ^28^ ^]^ Moreover, advances in 3D printing further enable the design and fabrication of personalized implants, customized to a patient's anatomy and biology, marking a significant advancement in personalized medicine.^[^ ^29^ ^]^

## 3 Regulatory Strategies and Biological Evaluation for the Development of Safe Vascular Medical Devices

The adoption of MEMS devices in cardiology, as all implantable vascular devices intended for long‐term in vivo applications, fulfills rigorous regulatory, biocompatibility, and biostability requirements. The development and clinical application of implantable vascular devices need careful integration of regulatory compliance, safety standards, and clinical best practices.

The approval process is subject to rigorous regulatory oversight and ensures that implantable medical devices are safe, effective, and traceable throughout their lifecycle.^[^ ^30^ ^]^ In the U.S., the Food and Drug Administration (FDA) regulates implantable devices under the Food, Drug, and Cosmetic Act (FD&C Act). It ensures that devices are safe and effective and directly supervises clinical investigations and postmarket reporting.^[^ ^31^ ^]^ For high‐risk devices, the Act requires manufacturers to obtain premarket approval before marketing, which involves submitting comprehensive preclinical and clinical data demonstrating the device's safety and effectiveness.^[^ ^32^ ^]^ MDR is one of the FDA's postmarket surveillance tools used to monitor device performance and assess long‐term safety, enabling the detection and management of rare, delayed or population‐specific risks.^[^ ^30^ ^]^

**Figure 2:**
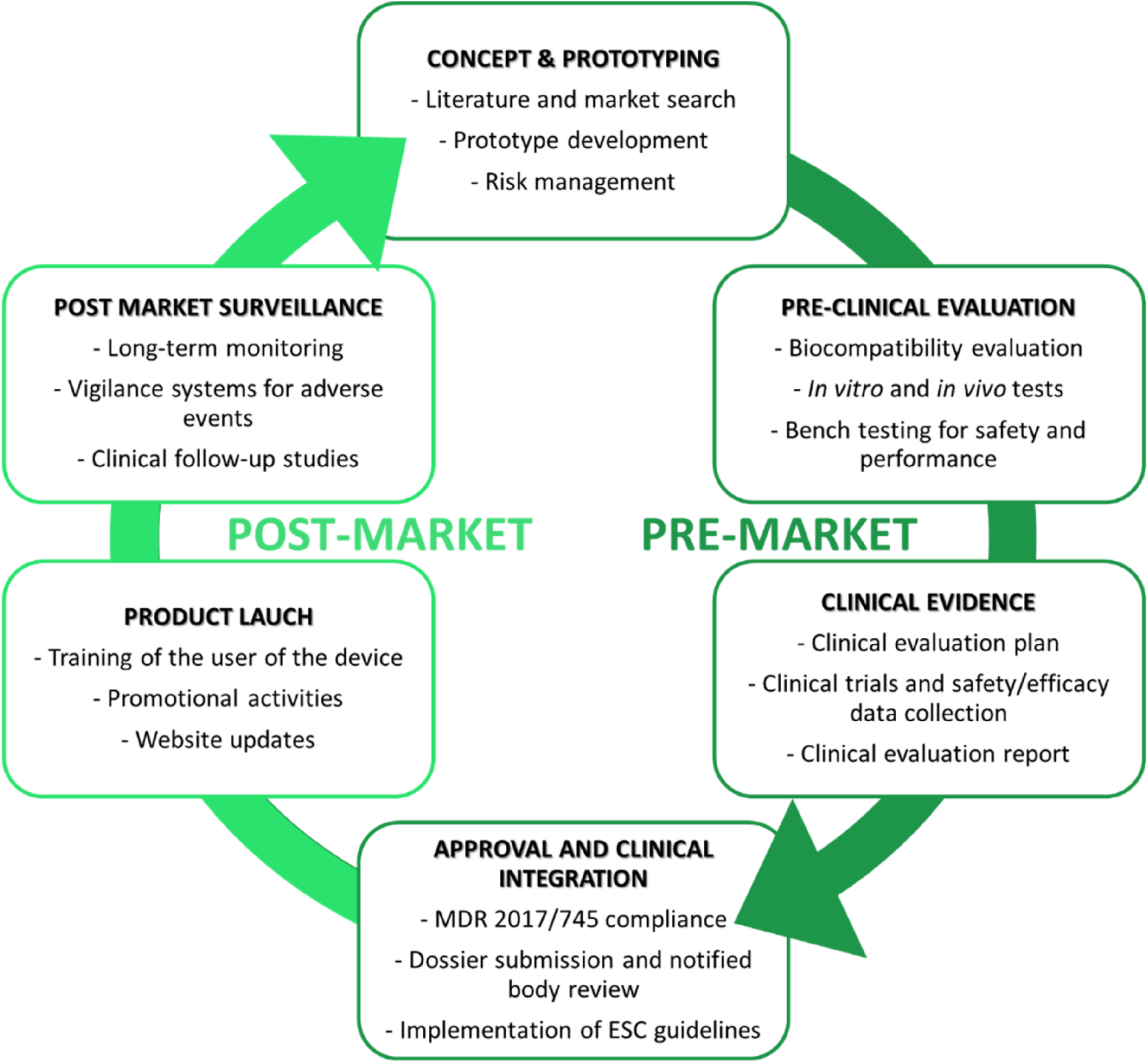
Medical device life cycle. The diagram outlines the main stages of developing a medical device life cycle, from initial concept and design, through prototyping and preclinical testing, to clinical evaluation, regulatory approval, and postapproval monitoring.

In the EU, the MDR provides the current legal framework for ensuring the safety, performance, and quality of medical devices throughout their life cycle.^[^ ^3^ ^]^ Replacing the former Medical Device Directive (MDD 93/42/EEC), the MDR introduced more rigorous requirements for preclinical evaluation, clinical evidence, risk management, and postmarket surveillance, especially for high‐risk, implantable devices that directly interact with the cardiovascular system (**Figure** **2**).

Under the MDR, implantable vascular devices are generally classified as class III (high risk) due to their invasive and long‐term use. About 40% of all new high‐risk medical devices are cardiovascular.^[^ ^33^, ^34^ ^]^ As a result, they are subject to stringent regulatory examination, including comprehensive preclinical and clinical assessments. Manufacturers must demonstrate biocompatibility, mechanical durability, and clinical safety while also providing evidence of long‐term performance through structured postmarket surveillance plans.^[^ ^35^, ^36^ ^]^ In the United Kingdom (UK), implantable medical devices are required to comply with the UK Medical Devices Regulations (UK MDR 2002) and to obtain certification under the UK Conformity Assessed marking scheme.^[^ ^37^ ^]^ In the EU and UK, notified bodies check and approve medical devices and conformity certificates before they can be sold. For some high‐risk devices, notified bodies must ask for advice from expert panels before giving the approval. These panels receive technical and scientific support from the European Medicines Agency.^[^ ^38^ ^]^ Notified bodies also ensure that devices meet safety, performance, and quality standards in alignment with the MDR and international standards set by the International Organization for Standardization (ISO). The British Standards Institution Assurance UK Ltd (BSI, Notified Body 0086) is currently the only UK‐approved body authorized to perform conformity assessments for these devices, ensuring their safety, performance, and compliance with regulations throughout their lifecycle.^[^ ^37^ ^]^ The ISO has produced the documentation collectively known as ISO 10993 that includes a series of rules dedicated to the biocompatibility of materials and devices.^[^ ^39^ ^]^ ISO 10993‐1 is often used in conjunction with ISO 13485 (quality management), ISO 14971 (risk management), and other standards, forming a comprehensive framework for ensuring the safety and regulatory compliance of medical devices. The certification process is highly resource‐intensive, often involving significant costs, long timelines, and the possibility of rejection or revision requests. This reflects the considerable complexity manufacturers face when bringing implantable devices to market.^[^ ^40^ ^]^

Another important goal is to provide timely access to innovative devices to benefit patient care. Complementing the regulatory landscape, ESC plays a pivotal role in defining cardiovascular clinical practice across Europe. Through its evidence‐based guidelines and recommendations, the ESC provides a framework for the appropriate use of implantable vascular devices in various clinical scenarios, such as acute coronary syndromes, peripheral artery disease, or congenital vascular malformations. While the ESC does not have regulatory authority, its guidelines influence clinical decision‐making and indirectly guide manufacturers in aligning device development with current therapeutic standards and unmet clinical needs. The goals of ESC are to establish priority access for unfulfilled devices for unmet needs and orphan cardiovascular medical devices, promote global regulatory alignment to simplify the approval process for cardiovascular medical devices across selected authorities, use early feasibility studies to evaluate initial safety and performance, and accelerate device development.^[^ ^41^ ^]^

Together, the MDR and ESC guidelines form a comprehensive network that governs not only the technical and regulatory aspects of vascular implantable devices but also their integration into routine patient care. This synergy ensures that devices brought to the market are not only compliant but also clinically relevant and effective.

The safety, performance, and quality of medical devices in the European market are ensured by ISO standards, such as ISO 14971, which outlines the identification and control of potential risks throughout their life cycle. Rigorous testing is a key component of regulatory submissions, providing evidence of safety, efficacy, and compliance with international guidelines. This comprehensive process is essential for market approval and helps minimize risks to patients and healthcare providers. Biocompatibility evaluation is an integral part of this process, spanning from initial design to postmarket surveillance. For this purpose, preclinical tests in vitro and ex vivo and animal studies are carried out to investigate the biological interactions following the device's implantation, to ensure compliance with current regulations, and to reduce potential risks. Implanted devices can trigger adverse biological responses, potentially leading to serious complications, compromised patency, or surgical failure, and ultimately reducing the implant's effectiveness and longevity.^[^ ^42^, ^43^, ^44^ ^]^

The tissue response to the implantation of a cardiovascular device is a complex and dynamic process, starts immediately upon contact between the device surface, blood, and vascular tissue. This response involves a tightly regulated sequence of biological events, inflammation, fibrosis, and thrombosis^[^ ^45^, ^46^ ^]^ aimed at restoring tissue integrity and maintaining homeostasis, but it can also lead to adverse outcomes that compromise device performance.^[^ ^47^ ^]^ Therefore, understanding and controlling this response is essential for improving cardiovascular implants’ biocompatibility and durability. These interactions pose significant challenges, highlighting the need for further innovations in biocompatible materials and device design.

To mitigate these challenges, it is essential to explore the biological events triggered by vascular‐device implantation. In the following sections, we will examine the intricate processes of inflammation and thrombosis that determine the fate of implantable vascular devices.

## 4 Vascular Responses to Implanted Devices: The Role of Inflammation and Thrombosis

**Figure 3:**
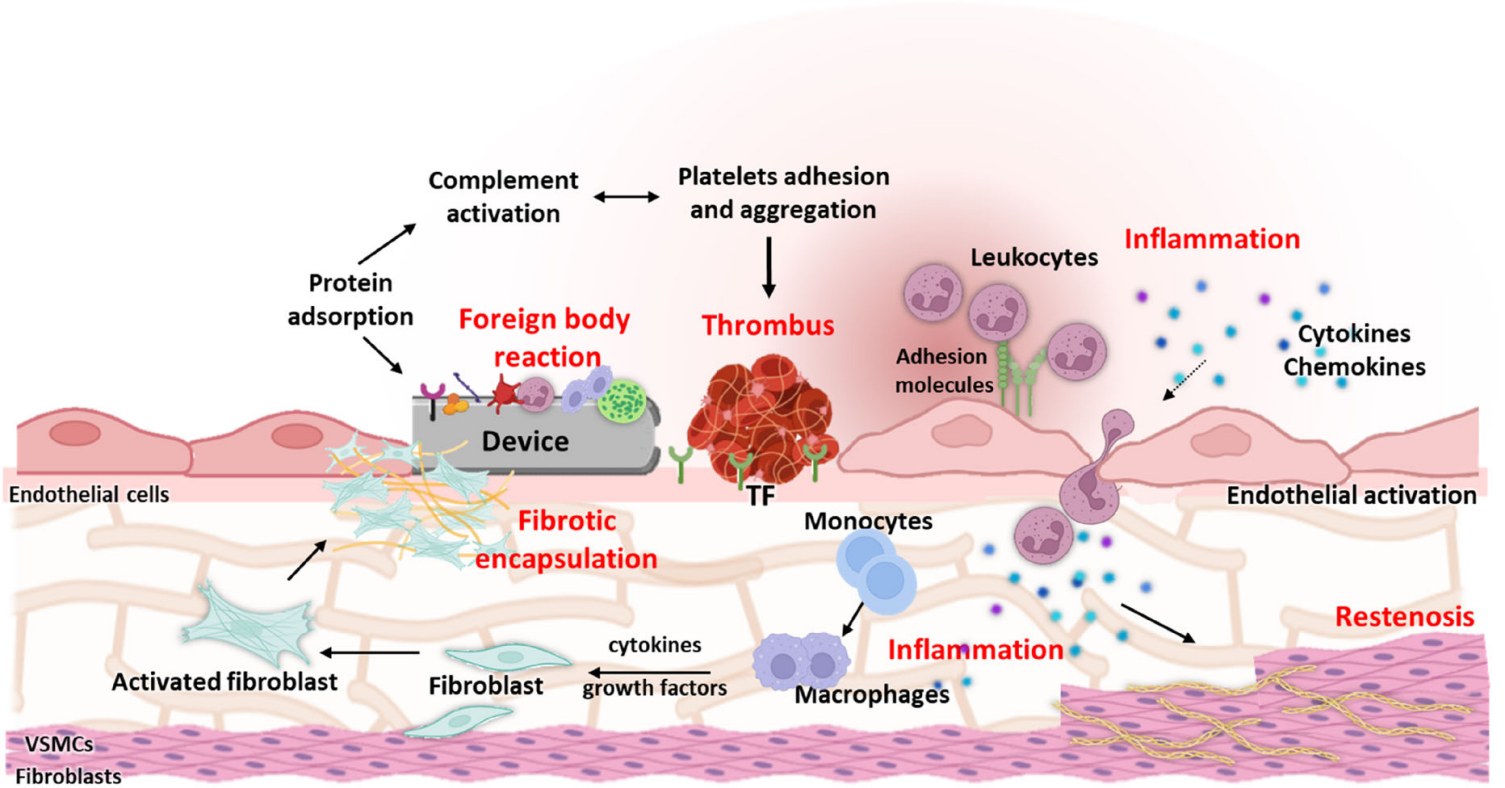
Schematic representation of the thrombo‐inflammatory response to device implantation. The device surface adsorbs plasma proteins, triggering the activation of immune cells (neutrophils and macrophages) and initiating the FBR, an acute and chronic inflammatory response triggered by the surface device, often resulting in the fibrosis and encapsulation of the implant. Activated endothelial cells promote platelet and leukocyte adhesion and transmigration by upregulation of adhesion molecules, cytokine release, and area of denudations expose subendothelial matrix components such as TF, promoting platelet adhesion, thrombus formation, and immune cell recruitment. In parallel, activation of abluminal fibroblasts and VSMCs promotes ECM deposition and the formation of a dense fibrotic capsule around the implant, impairing its function. VSMCs further contribute to neointimal hyperplasia and luminal narrowing, ultimately resulting in restenosis.

The vascular implantation of a medical device can trigger inflammation and thrombosis, a condition known as thrombo‐inflammation, resulting from the interplay between blood components and the device surface, as well as the activation of vascular wall cells (**Figure** **3**).^[^ ^5^, ^47^ ^]^ These mechanisms influence the host response to the implant and represent critical determinants of its biocompatibility, long‐term performance, and clinical efficiency. Once implanted, the device presents a bioactive surface to circulating blood components, facilitating immune cell adhesion and activation and initiating coagulation, contributing to vascular injury (Figure 3).^[^ ^5^ ^]^ Unlike artificial biomaterials, the endothelium, which lines the vessel and is directly exposed to the bloodstream, plays a physiological role in maintaining vascular homeostasis by producing antithrombotic and anti‐inflammatory molecules. Implanted medical devices can induce endothelial activation and compromise endothelial integrity, shifting the hemostatic balance toward a prothrombotic and proinflammatory state.

The proinflammatory response, initiated at the device‐blood interface, also extends to the abluminal side of the vessel wall, where fibroblasts and vascular smooth muscle cells (VSMCs) become activated during chronic inflammatory response. Activated fibroblasts increase the production of extracellular matrix (ECM) proteins, leading to the development of a dense fibrotic capsule around the device. isolating the implant from the surrounding tissue and ultimately compromising its long‐term performance. In parallel, VSMCs undergo phenotypic switching from a contractile to a synthetic and proliferative state, can migrate toward the intimal layer, and contribute to neointimal thickening.^[^ ^48^ ^]^ This process results in luminal narrowing and restenosis, further limiting the efficacy of the implanted device (Figure 3).

### 4.1 Device Surface‐ and Vascular Cell‐Mediated Inflammation

The body recognizes the implanted device surface as a foreign material, triggering the FBR. Upon contact with blood, the surface quickly adsorbs circulating plasma proteins, such as fibrinogen, albumin, complement proteins, fibronectin, and von Willebrand factor (vWF), forming a temporary matrix that supports cell accumulation and interaction with the foreign body.^[^ ^49^ ^]^ These proteins serve as ligands for receptors on platelets, leukocytes, and other immune cells, triggering the inflammation cascade and the coagulation.

Protein adsorption onto the biomaterial surface initiates the activation of the complement system, leading to the release of proinflammatory mediators such as complement factors C3a, C4a, and C5a. These molecules act as chemoattractants, promoting the recruitment of immune cells and amplifying the inflammatory response.^[^ ^33^, ^34^, ^50^ ^]^ Neutrophils are among the first responders, migrating to the site and releasing proteolytic enzymes and reactive oxygen species (ROS) to break down cellular debris and foreign material.^[^ ^33^ ^]^ Soon after, macrophages and undifferentiated monocytes accumulate in the surrounding tissue and device surface. In the presence of foreign implants, macrophages differentiate into classically activated proinflammatory macrophages (M1) that degrade materials through phagocytosis, release ROS and lysosomal enzymes, and alternatively activate prohealing macrophages (M2).^[^ ^51^, ^52^, ^53^, ^54^ ^]^

**Figure 4:**
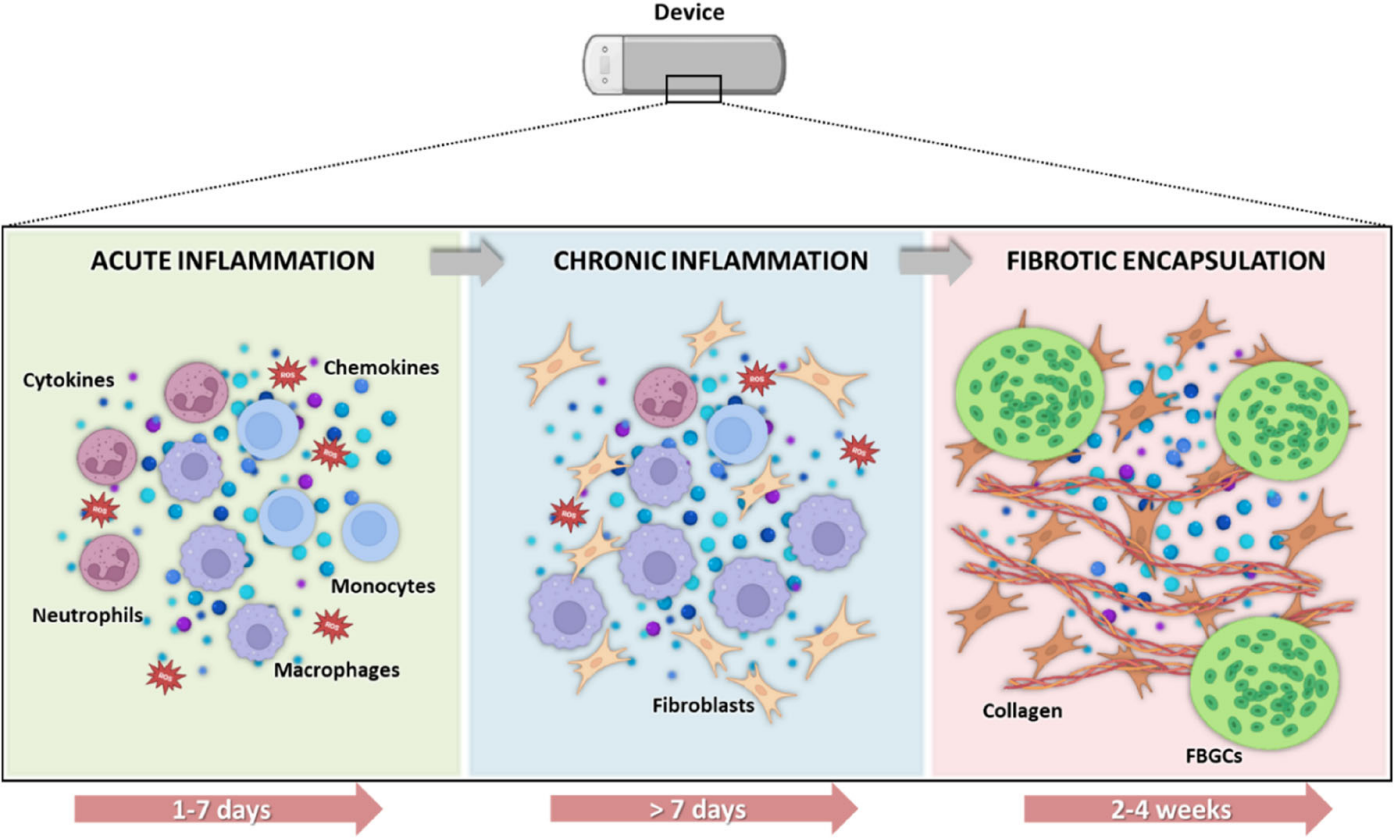
The stages of the FBR. FBR begins with acute inflammation involving neutrophils and macrophages, followed by chronic inflammation driven by persistent macrophage activity and fibroblast recruitment. This culminates in fibrotic encapsulation, where collagen and FBGCs isolate the implant. The timeline represents the typical progression of FBR following biomaterial implantation, including acute inflammation (1–7 days), chronic inflammation (>7 days), and fibrotic encapsulation (2–4 weeks).

Acute inflammation generally resolves within 3–7 days, depending on the immune system's capacity to control and resolve the response. However, if the inflammatory stimulus persists, chronic activation of macrophages and prolonged secretion of proinflammatory cytokines can persist for several weeks.^[^ ^55^, ^56^, ^57^, ^58^ ^]^ The chronic inflammatory reaction and the continuous proliferation of macrophages M1 at the implant site are the basis of the FBR (**Figure** **4**).^[^ ^59^ ^]^ Under the influence of cytokines, macrophages undergo fusion, forming multinucleated foreign body giant cells (FBGCs).^[^ ^60^, ^61^ ^]^ FBGC formation is associated with the release of lytic enzymes as matrix metalloproteinases, and ROS, which contribute to ECM degradation and thickening of the vessel wall.^[^ ^62^ ^]^ As the inflammatory response persists, FBGCs secrete profibrotic cytokines and growth factors, which promote the migration of fibroblasts from the surrounding adventitial tissue to the implant surface, where they contribute to ECM deposition, primarily consisting of collagen and fibronectin.^[^ ^63^ ^]^ Under inflammatory stimuli, proinflammatory macrophages can also differentiate into myofibroblasts, which generate contractile forces and contribute to fibrotic capsule formation.^[^ ^64^, ^65^ ^]^ This leads to the formation of fibrous tissue that ends up encapsulating the implant, potentially impairing its functionality by isolating it from surrounding tissues (Figure 4).^[^ ^63^, ^66^ ^]^ In parallel, fibrosis is further amplified by the activation of CD4^+^ and CD8^+^ T cells, which release proinflammatory cytokines that enhance fibroblast collagen production. B cells also contribute by forming immune complexes on the surface of the device, which intensify chronic inflammation.^[^ ^60^, ^67^ ^]^

In parallel with the inflammatory response at the biomaterial–tissue interface, the surrounding endothelium actively contributes to regulating both the initial inflammation and its subsequent progression toward fibrosis. Altered shear stress caused by the presence of the device, degradation products of biomaterials, and proinflammatory molecules released by cell activation on the biomaterial surface contribute to disrupting vascular homeostasis, promoting endothelial activation, inflammation, and hindering tissue integration of the implant. Endothelial activation is characterized by reduced secretion of key endothelium‐derived vasoactive molecules such as nitric oxide (NO), increased release of proinflammatory mediators, and upregulation of adhesion molecules, which facilitate leukocyte recruitment and transmigration through the endothelium (or its remnants), accumulating at the implantation site and contributing to both acute and chronic inflammatory responses.^[^ ^68^ ^]^ When a medical device is implanted, it exerts persistent mechanical stress at the implantation site from the pressure against vessel walls, pulsatile blood flow, or the device's movement and vibration, which can compromise the structural integrity of endothelial cells.^[^ ^69^ ^]^ These areas of endothelial denudation promote the recruitment of immune cells and the adhesion and activation of platelets at the site, intensifying inflammation and exposing the subendothelial matrix, which is rich in collagen and tissue factor (TF). These potent activators of platelet aggregation and the coagulation cascade are also present.^[^ ^70^ ^]^

While fibroblasts are the principal mediators of fibrous capsule formation around vascular implants, VSMCs may contribute to peridevice tissue remodeling and neointimal thickening within the vessel wall.^[^ ^71^ ^]^ Sustained inflammation at the device–tissue interface, driven by activated endothelial cells and recruited macrophages, triggers VSMC activation, characterized by a phenotypic switch from a contractile to a synthetic and migratory state. Activated VSMCs respond to cytokines, chemokines, and mechanical stimuli such as stretch or altered flow, further amplifying the local inflammatory milieu. They migrate from the medial layer to the intima, where they proliferate and secrete ECM components, including collagen and fibronectin.^[^ ^72^ ^]^ The accumulation of VSMCs and ECM leads to neointimal formation, progressively narrowing the vascular lumen and contributing to restenosis, which can ultimately result in vessel occlusion.^[^ ^72^ ^]^ These processes compromise long‐term device patency and frequently necessitate additional clinical interventions or device replacement.

### 4.2 Thrombogenic Mechanisms at the Vascular–Device Interface

**Figure 5:**
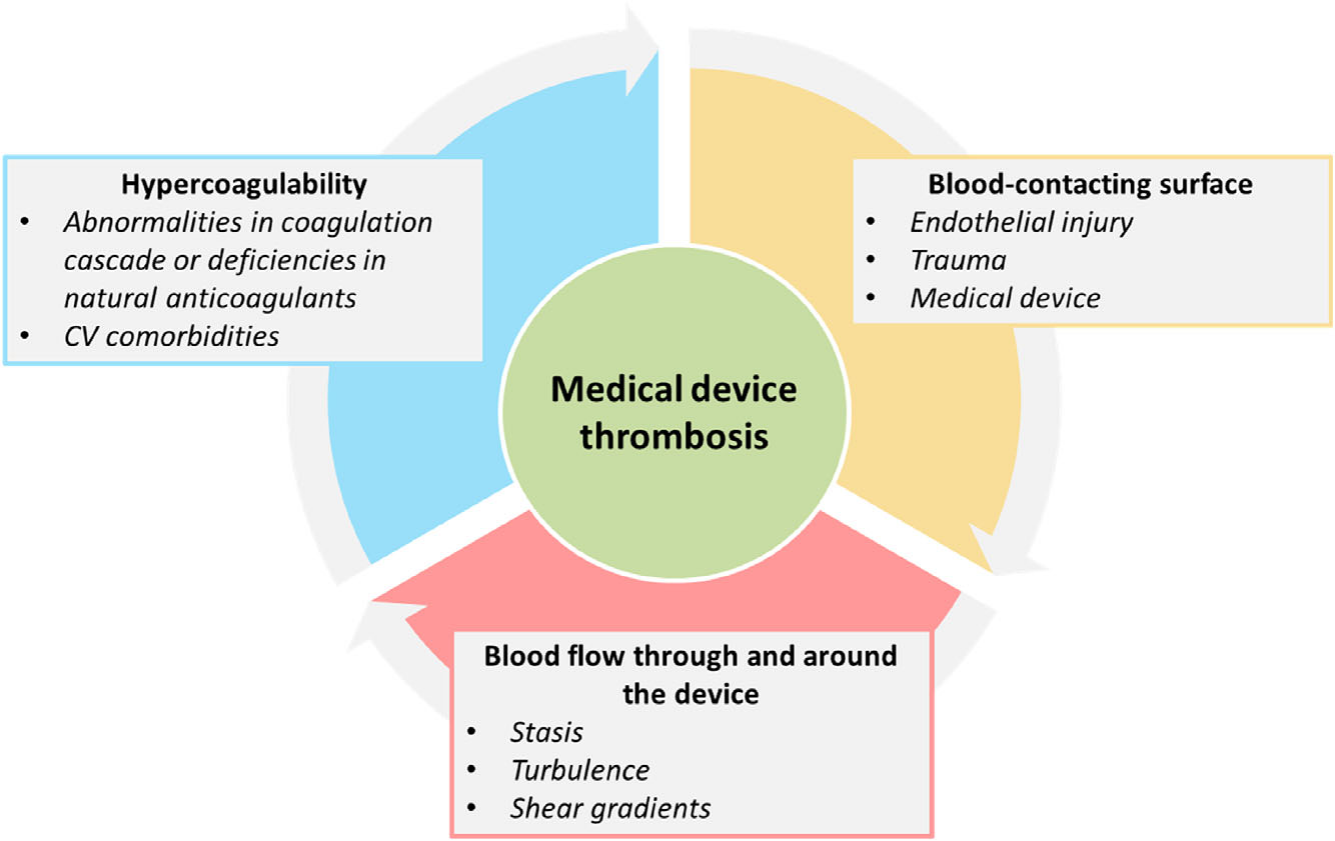
Virchow's triad in medical device‐related thrombosis. A modified version of Virchow's triad explains the mechanisms underlying device‐related thrombosis through three main factors: hypercoagulability, blood‐contacting surfaces (device surface, endothelial injury), and abnormal flows. CV.

All implanted materials or devices within the human cardiovascular system are inherently thrombogenic and can perturb hemostasis. The formation of blood clots at the implant site is a common cause of failure of these devices and can obstruct blood flow, leading to serious issues like ischemia, stroke, or organ damage.^[^ ^73^, ^74^ ^]^ Consequently, patients typically require short‐ or long‐term anticoagulant therapy to mitigate the risk of device‐associated thrombosis and ensure the long‐term functionality and safety of the implant. Device‐related thrombosis can result from a complex interplay of factors, including patient‐specific conditions, clinical management practices, and the device's inherent characteristics. Specifically, the mechanisms underlying thrombosis triggered by implantable vascular devices can be elucidated through a modified version of Virchow's triad that includes three key determinants of thrombogenesis: hypercoagulability, blood‐contacting surface, and aberrant flow (**Figure** **5**).^[^ ^4^ ^]^

#### 4.2.1 Hypercoagulability

The hypercoagulable condition of patients can further increase the risk of thrombus formation. Patient‐related factors include inherent risks such as a hypercoagulable state, infections, vascular disorders, or endothelial dysfunction. Primary hypercoagulability is caused by genetic mutations or inherited conditions, typically due to abnormalities in the clotting cascade or deficiencies in natural anticoagulants (i.e., factor V Leiden, protein C/S deficiency).^[^ ^75^, ^76^ ^]^ Secondary hypercoagulable states often result from underlying diseases (e.g., malignancies, systemic lupus erythematosus, or nephrotic syndrome), lifestyle changes, or environmental exposures. Clinical factors in the patient's care, such as incorrect device positioning, device kinking, or insufficient anticoagulation therapy, can also elevate the risk of thrombosis.^[^ ^75^, ^76^ ^]^

#### 4.2.2 Endothelial‐ and Device Surface‐Mediated Thrombosis

The risk of thrombosis after the implantation of an intravascular medical device also depends on the properties of the blood‐contacting surfaces, such as the endothelium and the device surface. Under physiological conditions, blood vessels maintain a tightly regulated hemostatic balance that preserves blood fluidity and enables prompt repair after injury. Disruption of this balance can lead to intravascular thrombosis and vessel occlusion. The activation of coagulation is the process leading to thrombus formation. It involves two interconnected pathways, the intrinsic and the extrinsic, both contributing to biomaterial‐associated thrombosis. The extrinsic pathway is initiated by damage to endothelial cells, whereas the intrinsic (or contact activation) pathway is triggered by interactions between the biomaterial surface and adsorbed plasma proteins.^[^ ^77^ ^]^

The endothelial cells respond to the surrounding milieu by multiple mechanisms to regulate thrombosis. Under physiological conditions, they produce anticoagulant, antifibrinolytic, and anti‐inflammatory molecules that prevent the activation of platelets and the coagulation cascade, maintaining blood fluidity through several fibrinolytic and antithrombotic processes.^[^ ^6^, ^48^ ^]^ Following vascular insult, the endothelium shifts from an anticoagulant to a procoagulant/prothrombotic phenotype, initiating numerous mechanisms to induce clotting (e.g., by expression of TF) and anticoagulation (e.g., by expression of thrombomodulin and PAR‐type receptors) to provide controlled vascular hemostasis at sites of injury.

When the endothelial monolayer is damaged, underlying matrix proteins such as collagen and vWF, are exposed to circulating blood and serve as critical substrates for platelet adhesion.^[^ ^78^ ^]^ A crucial step in this process is the binding of fibrinogen to the platelet integrin αIIbβ3, which stabilizes platelet adhesion and promotes their aggregation.^[^ ^79^ ^]^ Activated platelets then release prothrombotic mediators that amplify the response by recruiting and activating additional platelets. This cascade results in the formation of a primary hemostatic plug, which temporarily occludes the vascular injury site and helps minimize blood loss.^[^ ^79^ ^]^ Once a platelet plug has been formed, the coagulation cascade is activated to stabilize this plug through the generation of a fibrin mesh. Upon vascular injury, exposure of subendothelial TF, normally not exposed to blood, triggers the extrinsic pathway of coagulation, by binding factor VII. The TF–VIIa complex activates factors IX and X, ultimately promoting thrombin generation.^[^ ^80^ ^]^ Thrombin is the key enzyme since it cleaves fibrinogen into fibrin. Fibrin polymerizes into a fibrin network, which forms the blood clot with the aggregating platelets. Furthermore, thrombin promotes platelet activation and, through positive feedback, activates the intrinsic pathway while through negative feedback, it activates activated protein C, an anticoagulant and profibrinolytic protein.^[^ ^80^ ^]^

In the absence of endothelial denudation, TF can be expressed on the surface of endothelial cells and monocytes under proinflammatory conditions.^[^ ^81^, ^82^ ^]^ As coagulation is activated, downstream factors like FVIIa and FXa, thrombin, fibrin fragments, are generated, initiating a feedback loop in which inflammation increases TF expression, driving more coagulation and amplifying the inflammatory response.

The vascular endothelial surface is covered by the glycocalyx matrix, a dynamic, carbohydrate‐rich layer composed of glycoproteins, proteoglycans, and glycosaminoglycans.^[^ ^83^ ^]^ This structure plays a critical vascular protective role, contributing to the regulation of vascular permeability, mechanotransduction and thrombosis prevention by inhibiting platelet adhesion and modulating the local balance of coagulation factors.^[^ ^83^ ^]^ The implantation of medical devices into the vasculature can mechanically damage the glycocalyx, reducing its thickness and integrity and compromising its antithrombotic functions.^[^ ^84^ ^]^ As a consequence, the underlying endothelial surface becomes more adhesive to platelets and leukocytes and the local equilibrium between pro‐ and anticoagulant factors is altered, favoring thrombus formation.^[^ ^85^ ^]^

**Figure 6:**
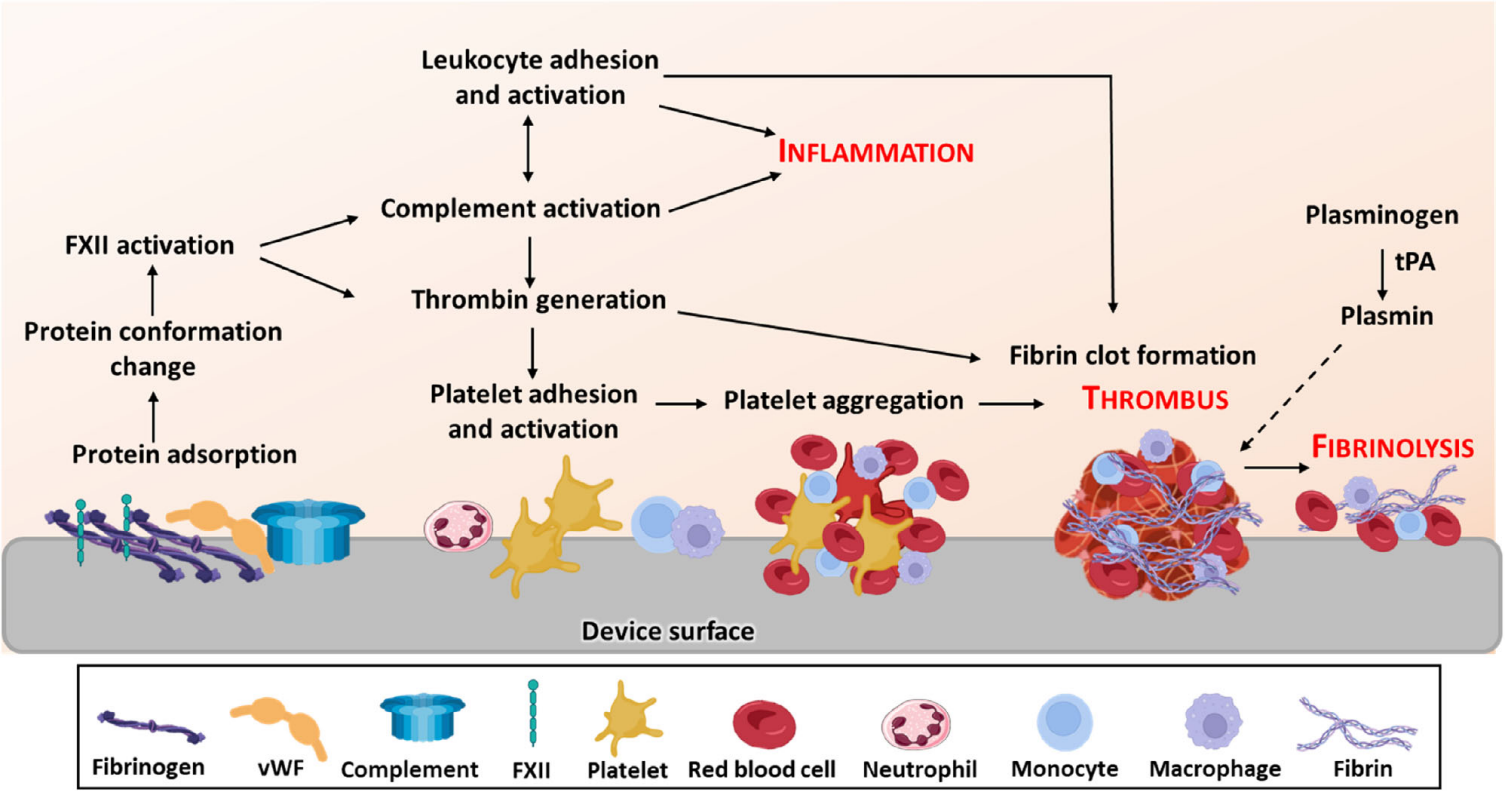
Schematic representation of thrombus formation on a medical device surface. Protein adsorption, including fibrinogen, vWF, complement components and factor XII (FXII), triggers a cascade of events: platelet adhesion and activation, with release of agonists that enhance aggregation; activation of the complement system, which amplifies platelet and leukocyte activation and promotes inflammation; and FXII initiating the intrinsic coagulation pathway, leading to fibrin clot formation and thrombus development. Plasmin, generated from plasminogen by tissue plasminogen activator (tPA), degrades fibrin (fibrinolysis), and restores normal blood flow.

Unlike the endothelium, the medical device surface promotes thrombosis involving nonspecific adsorption of proteins, complement activation, adhesion of platelets, leukocytes and red blood cells, and thrombin generation (**Figure** **6**).^[^ ^5^, ^86^, ^87^ ^]^

Negatively charged or hydrophilic biomaterial surface components can activate the contact system, thereby facilitating the activation of the intrinsic pathway of coagulation. Proteins such as fibrinogen, fibronectin, complement proteins, plasma Prekallikrein (PK), coagulation factors (XI (FXI) and FXII/XIIa), and vWF adsorb to biomaterial surfaces. The complement system activation at the implantation site promote activation of the intrinsic pathway of coagulation, the platelet adhesion and activation, inflammatory response and the expression of TF on circulating monocytes, thereby amplifying the coagulation cascade.^[^ ^5^, ^88^ ^]^ After adhesion, platelets become activated and release agonists that amplify adhesion, activation, and aggregation on the artificial surface, leading to platelet thrombus formation. Adsorbed factor XII is autoactivated to FXIIa, which converts PK into kallikrein, further activating more FXII and creating a positive feedback loop. Activation of FXI triggers the intrinsic pathway of coagulation, culminating in thrombin generation, which induces platelet‐fibrin clot formation on the biomaterial surface. In addition, FXIIa induces complement activation.^[^ ^5^ ^]^

The complement system and intrinsic pathway are two interconnected mechanisms involved in the initiation of clotting on blood‐contacting medical devices. Activation of the complement cascade enhances thrombosis by promoting platelet adhesion and activation, amplifying the coagulation cascade, inflammatory response, and the expression of TF on circulating monocytes, thereby amplifying the coagulation cascade.^[^ ^5^, ^88^, ^89^ ^]^

Leukocytes, particularly neutrophils, adhere to adsorbed fibrinogen via an integrin complex. Adherent platelets promote leukocyte adhesion via an interaction between P‐selectin on the surface of activated platelets and leukocyte P‐selectin glycoprotein ligand, as do activated complement components. In addition to generating superoxide and other free radicals, adherent leukocytes may release platelet‐activating factor and proinflammatory cytokines, which amplify the inflammatory response, leading to further recruitment of immune cells to the site.^[^ ^90^ ^]^

Adherent red cells can release adenosine diphosphate, which activates platelets and under high‐shear conditions, erythrocyte hemolysis may occur.^[^ ^91^ ^]^

An important pathway to consider in the context of coagulation–implanted devices is fibrinolysis, the process responsible for clot degradation. This mechanism is regulated by the protease tissue‐type plasminogen activator, which catalyzes the conversion of plasminogen into plasmin, the enzyme that cleaves and degrades the fibrin mesh within the clot.^[^ ^74^ ^]^ The fibrinolytic system supports device functionality by breaking down fibrin and maintaining balanced fibrinolytic activity essential for the long‐term performance and safety of implantable vascular devices. It ensures proper blood flow around the implant, reduces the risk of thromboembolic events, and contributes to the overall biocompatibility of the device.

#### 4.2.3 Flow Disturbances Induced by Vascular Devices

Understanding the vessel type and flow dynamics is essential when implanting a medical device to ensure correct functionality, minimize complications, and promote long‐term vascular health. The structure of the blood vessel wall plays a crucial role in regulating vascular function and responding to mechanical forces, such as shear stress, the frictional force exerted by blood flow on the vessel walls. The ability of the endothelium to adapt its phenotype to altered flow conditions is critical for maintaining vascular health after the implantation of medical devices.^[^ ^7^, ^92^, ^93^ ^]^ Under normal conditions, high‐shear stress laminar flow creates a uniform high‐shear stress on endothelial cells, promoting endothelial protective and anti‐inflammatory responses, while low or oscillatory shear stress does not.^[^ ^94^ ^]^

In arteries, higher shear stress promotes endothelial cell alignment, a process crucial for maintaining vessel health. Arteries are thicker, with more smooth muscle and experience higher pressure due to the heart's pumping action, leading to pulsatile blood flow and varying levels of shear stress on endothelial cells.^[^ ^12^, ^95^ ^]^ The placement of a device in an artery must consider these fluctuations in flow, as they can affect how well the device integrates with the vessel. Conversely, in veins, lower shear stress leads to a different endothelial phenotype, which may be more sensitive to changes in pressure and flow.^[^ ^95^ ^]^ Veins have thinner walls, less smooth muscle, and lower pressure, resulting in slower, more laminar flow with less intense shear stress on the endothelium. Devices implanted in veins should be designed to adapt to the lower pressure and more gradual flow. If placed inappropriately, devices could alter the vein's ability to return blood to the heart, potentially causing long‐term issues. This lower shear stress leads to a different endothelial phenotype, more responsive to changes in pressure or flow.

However, when a medical device is introduced, abnormal or fluctuating shear stress, regardless of vessel type, can lead to endothelial injury, inflammation, and thrombosis, contributing to the development and progression of cardiovascular conditions. The device can create turbulence in the cardiovascular system, producing areas of low endothelial shear stress and local flow recirculation in the region of proximal and distal sites of the device, where procoagulant and proinflammatory factors can accumulate, leading to endothelial dysfunction and increasing the risk of thrombosis or restenosis.^[^ ^96^, ^97^ ^]^ Conditions of high‐shear stress can additionally contribute to thrombogenesis through hemolysis, where heme released by lysed red blood cells is catabolized to carbon monoxide, implicated in enhancing clot strength and the velocity of thrombus growth promoting hyperfibrinogenemia and impairing fibrinolysis.^[^ ^98^ ^]^ Moreover, disturbed laminar blood flow can impede the reendothelialization, critical for healing and restoring normal vascular function.^[^ ^99^ ^]^

The glycocalyx is highly sensitive to hemodynamic forces, particularly the shear stress generated by blood flow. Abnormal blood shear stress has been shown to increase glycocalyx shedding in cultured human vascular endothelial cells.^[^ ^85^ ^]^ Intravascular devices often alter these forces by inducing turbulent flow and regions of abnormally low or high‐shear stress.^[^ ^100^ ^]^ These altered flow patterns not only cause further mechanical damage to the glycocalyx but may also impair its regeneration and maintenance, perpetuating endothelial dysfunction and increasing the risk of thrombosis over time. To mitigate these adverse effects, recent advancements have focused on developing glycocalyx‐mimetic coatings for medical devices. For instance, a study demonstrated that a glycocalyx‐like multifunctional coating on titanium surfaces significantly improved hemocompatibility and cytocompatibility, suggesting a promising approach to enhance the integration and performance of implantable devices.^[^ ^84^, ^101^ ^]^

## 5 Biomaterials and Surface Engineering Strategies

On the biological side, the implantation of a device in the vascular wall and its degradation byproducts can trigger thrombosis and immune responses, which in turn may compromise long‐term device function, contribute to implant degradation and impact patient outcomes.^[^ ^43^, ^44^, ^45^, ^60^, ^63^ ^]^ Consequently, there is increasing interest in the design, development of new biomaterials that modulate the vascular microenvironment.

Historically, textile materials like Dacron (polyethylene terephthalate) have been the standard for vascular grafts, particularly for large‐diameter vessels, due to their excellent mechanical strength, long‐term stability, and ease of suturing.^[^ ^102^ ^]^ However, Dacron is associated with a high risk of thrombosis and induces an early inflammatory response.^[^ ^4^ ^]^ Similarly, the polymer expanded polytetrafluoroethylene has been widely used for vascular grafts because of its controlled porosity and good blood compatibility, which reduces platelet adhesion and provides good flexibility and high tensile strength.^[^ ^103^ ^]^ Despite these advantages, its limited capacity for spontaneous endothelialization can lead to intimal hyperplasia.^[^ ^104^, ^105^ ^]^

Metallic materials, such as titanium and nitinol, are widely used for stents. Titanium offers excellent corrosion resistance, high mechanical strength, and good biocompatibility.^[^ ^106^ ^]^ Nitinol, a nickel–titanium alloy known for its super elasticity and shape memory properties, is ideal for adapting to dynamic vessel motion.^[^ ^107^ ^]^ However, the surfaces of both metals are inherently thrombogenic, triggering coagulation and platelet adhesion, and therefore require lifelong antiplatelet therapy to prevent thrombosis.^[^ ^108^, ^109^ ^]^

To address these challenges, advanced surface engineering strategies are being explored to enhance hemocompatibility and reduce immune activation. These approaches include plasma surface activation, heparin immobilization, and drug‐eluting coatings, such as hydrophobic multilayers of Duraflo heparin and sirolimus, which significantly reduce protein adsorption, platelet adhesion and inflammatory responses, while enabling controlled, site‐specific drug delivery.^[^ ^110^, ^111^, ^112^ ^]^ Moreover, the most promising developments are polymer brush‐based coating, such as biomaterials incorporating poly(ethylene glycol) (PEG) or zwitterionic surfaces, which offer strong antifouling properties by resisting protein adsorption and platelet attachment.^[^ ^113^ ^]^ These polymer architectures form stable brush‐like layers that strongly bind to the substrate, repelling plasma proteins and bacteria and reducing thrombogenicity and inflammatory responses.^[^ ^114^ ^]^

Surface topography also plays a critical role in modulating inflammation: high surface‐to‐volume materials like porous scaffolds recruit more macrophages and FBGCs, while smooth surfaces are more prone to fibrosis.^[^ ^58^ ^]^ Nano‐ and micropatterning techniques create controlled surface topographies on the nano‐ or microscale and have emerged as powerful tools to modulate their adhesion, morphology, and activation state of macrophages and fibroblasts.^[^ ^115^, ^116^ ^]^ Additionally, surfaces that mimic the endothelial glycocalyx have shown improved hemocompatibility and reduced platelet activation, thereby supporting long‐term device patency, vascular homeostasis, and a reduction in systemic complications.^[^ ^85^ ^]^ A glycocalyx‐like multifunctional coating on titanium surfaces significantly improved hemocompatibility and cytocompatibility, suggesting a promising approach to enhance the integration and performance of implantable devices.^[^ ^84^, ^101^ ^]^

More advanced biomaterials aim to replicate the dynamic biological behavior of the endothelium. These functionalized surfaces are capable of releasing NO, responding to shear stress, presenting anti‐inflammatory signals, and recruiting endothelial progenitor cells.^[^ ^117^ ^]^ They can inhibit macrophage activation, reduce neutrophil recruitment, and enhance endothelial integration.^[^ ^111^ ^]^

In this context, biomaterials engineered to release proendothelial growth factors or to present endothelium‐specific adhesion peptides can support reendothelialization and the restoration of monolayer integrity.^[^ ^118^ ^]^ Furthermore,^[^ ^119^ ^]^ stimuli‐responsive materials, activated by pH, oxidative stress or enzymatic activity, allow for precise, localized delivery of therapeutic agents, while^[^ ^120^ ^]^ simultaneously modulating the immune response and promoting vascular regeneration.^[^ ^119^, ^120^ ^]^ These multifunctional platforms represent an innovative, synergistic approach that targets multiple thrombogenic pathways simultaneously, effectively reducing local thrombosis, minimizing side effects and enhancing long‐term device performance.^[^ ^85^ ^]^ Additionally, emerging systems as nanostructured surfaces, such as polycaprolactone‐based substrates and microgrooved polymers, have shown promise in promoting hemocompatibility and reendothelialization.^[^ ^117^, ^121^ ^]^ Nanoparticle‐based delivery systems are being developed to carry thrombolytic or anti‐inflammatory agents directly to the site of thrombosis, with potential for real‐time imaging.^[^ ^122^, ^123^ ^]^

Inflammation and coagulation are not the only biological effects that can occur following the implantation of a medical device. Device‐associated infections represent a significant clinical complication, often resulting from the ability of bacteria to adhere to biomaterial surfaces and form biofilms.^[^ ^124^ ^]^

Microorganisms adhere to the device surface, produce extracellular polymeric substances and form mature biofilms characterized by increased resistance to host immune defenses and antimicrobial therapies.^[^ ^125^ ^]^ Despite mechanisms such as phagocytosis, ROS generation and extracellular trap formation, neutrophils and macrophages exhibit reduced efficacy against biofilm‐embedded bacteria,^[^ ^126^ ^]^ favoring chronic inflammation and tissue damage.^[^ ^127^ ^]^

The physicochemical characteristics of biomaterial surfaces, including chemical composition, wettability, surface charge, topography, roughness, and stiffness, may influence bacterial adhesion and affect implant‐associated infection risk and overall clinical performance.

Considerable efforts have been focused on the development of antiadhesive coatings, such as hydrophilic polymers or PEG, which minimize nonspecific protein adsorption and bacterial attachment.^[^ ^128^ ^]^ Cationic polymers, such as carboxymethyl chitosan and pristine chitosan, act as antimicrobial agents.^[^ ^129^, ^130^ ^]^

Nanomaterials have been employed to confer bactericidal activity at the device interface due to their tunable physicochemical properties, high surface‐to‐volume ratio and potential for functionalization with antimicrobial agents.^[^ ^131^ ^]^ Zinc oxide and silver nanoparticles (AgNPs) have been proposed as excellent antimicrobial agents, acting through direct interactions with membranes and DNA and by inducing ROS that disrupt the cell membrane.^[^ ^132^, ^133^ ^]^ Titanium‐made implants coated with copper‐containing nanoparticles reduce the requirement for prophylactic antibiotics.^[^ ^134^ ^]^

Enzymes such as haloperoxidases, myeloperoxidases, and lactoperoxidases have been utilized as antimicrobial implant coatings, exploiting hydrogen peroxide to generate reactive species that disrupt bacterial membranes and proteins.^[^ ^135^ ^]^

Overall, progress in biomaterials and surface engineering has significantly enhanced the ability to address thrombosis, inflammation, and infection in vascular implants. Despite these advancements, the long‐term degradation of implantable materials remains a major concern. Prolonged exposure to the chemically reactive and mechanically dynamic in vivo environment can undermine the stability and function of even the most advanced biomaterials, leading to the breakdown of protective coatings and loss of insulation in electronic parts. The degradation mechanisms of implantable devices are complex and depend on both material properties and the surrounding biological environment. Major processes include oxidation, hydrolysis, mechanical stress, enzymatic activity from adjacent tissues, and the action of phagocytic cells, all of which can weaken the structural and functional integrity of the device over time.^[^ ^136^ ^]^ Therefore, future materials strategies must balance device functionality with durability, stability, and controlled degradation to ensure safe and reliable long‐term performance.

## 6 Conclusions

The development of implantable vascular devices has transformed the management of cardiovascular diseases by enabling more effective diagnosis, treatment, and patient monitoring. However, their clinical translation faces both regulatory and biological challenges.

From a regulatory perspective, authorities such as the EU MDR and the U.S. FDA impose stringent requirements to ensure the safety, effectiveness, clinical performance, and quality management of medical devices before market approval. While rigorous clinical trials and comprehensive safety assessments are essential to protect patients, these processes are inherently complex, time‐consuming, and costly, often slowing innovation. Despite continuous advances in device design and preclinical testing, navigating such demanding regulatory pathways remains one of the major challenges in translating promising technologies from bench to bedside, highlighting the need for strategic planning and early engagement with regulatory authorities.

The biological response to vascular implants can significantly influence their long‐term success. Inflammatory reaction, thrombus formation, fibrotic tissue development, and impaired endothelial regeneration are common complications that may compromise device functionality and worsen clinical outcomes.

Next‐generation devices must incorporate innovative biomaterials and engineered surfaces to better manage thrombogenicity, immune responses, endothelial healing, and infection risk. At the same time, these innovations must align with the growing demand for cost‐effective solutions that ensure equitable access and personalized therapies that adapt to the unique biological and clinical profiles of each patient. By combining technological advances with evidence‐based health policies, implantable devices can support a care model that is both clinically effective and financially sustainable.

Advancing implantable medical devices requires a multidisciplinary approach—combining biomedical engineering, material science, vascular biology, clinical medicine, and regulatory frameworks along with systems capable of translating device‐generated data into personalized, timely therapeutic interventions.

## Conflict of Interest

The authors declare no conflict of interest.
